# Publisher Correction: Z-ring membrane anchors associate with cell wall synthases to initiate bacterial cell division

**DOI:** 10.1038/s41467-019-08410-y

**Published:** 2019-01-30

**Authors:** Manuel Pazos, Katharina Peters, Mercedes Casanova, Pilar Palacios, Michael VanNieuwenhze, Eefjan Breukink, Miguel Vicente, Waldemar Vollmer

**Affiliations:** 1 Centre for Bacterial Cell Biology, Institute for Cell and Molecular Biosciences, Newcastle University, Richardson Road, Newcastle upon Tyne, NE2 4AX UK; 2 Centro Nacional de Biotecnología-Consejo Superior de Investigaciones Científicas (CNB-CSIC), Darwin 3, 28049 Madrid, Spain; 3 Molecular and Cellular Biochemistry Department, Biology Department, Indiana University, 212S. Hawthorne Dr, Bloomington, IN 47405 USA; 4 Membrane Biochemistry and Biophysics, Bijvoet Center for Biomolecular Research, Department of Chemistry, Faculty of Science, Utrecht University, Padualaan 8, 3584 CH Utrecht, The Netherlands

Correction to: *Nature Communications*; 10.1038/s41467-018-07559-2; published online 30 November 2018

The original version of this Article contained errors in Figs. 1 and 3. In Fig. 1b, the label ‘IP-PBP3’ above the second of the three blots incorrectly read ‘IP-PBP1B’. In Fig. 3b, the label ‘PBP1B’ under the first bar of each chart incorrectly read ‘PBP1A’. These errors have been corrected in both the PDF and HTML versions of the Article.

