# Supplementary material for: Z-ring membrane anchors associate with cell wall synthases to initiate bacterial cell division
Source: Nat Commun. 2018 Nov 30;9:5090. doi: 10.1038/s41467-018-07559-2 (PMC6269477; doi:10.1038/s41467-018-07559-2)
Supplement: Supplementary file 1 — Supplementary Information [file 41467_2018_7559_MOESM1_ESM.pdf]

SUPPLEMENTAL INFORMATION

Z-ring membrane anchors associate with cell wall synthases to initiate bacterial cell division

Manuel Pazos[1], Katharina Peters[1], Mercedes Casanova[2], Pilar Palacios[2], Michael VanNieuwenhze[3], Eefjan Breukink[4], Miguel Vicente[2], Waldemar Vollmer[1*]

[1] Centre for Bacterial Cell Biology, Institute for Cell and Molecular Biosciences, Newcastle University, Richardson Road, Newcastle upon Tyne, NE2 4AX, UK.

[2] Centro Nacional de Biotecnología- Consejo Superior de Investigaciones Científicas (CNB-CSIC), Darwin 3, Madrid 28049, Spain.

[3] Molecular and Cellular Biochemistry Department, Biology Department, Indiana University, 212 S. Hawthorne Dr, Bloomington, 47405 Indiana, USA.

[4] Membrane Biochemistry and Biophysics, Bijvoet Center for Biomolecular Research, Department of Chemistry, Faculty of Science, Utrecht University, Padualaan 8, 3584 CH, Utrecht, The Netherlands.

* Correspondence and requests for material should be addressed to W.V. (email: w.vollmer@ncl.ac.uk)

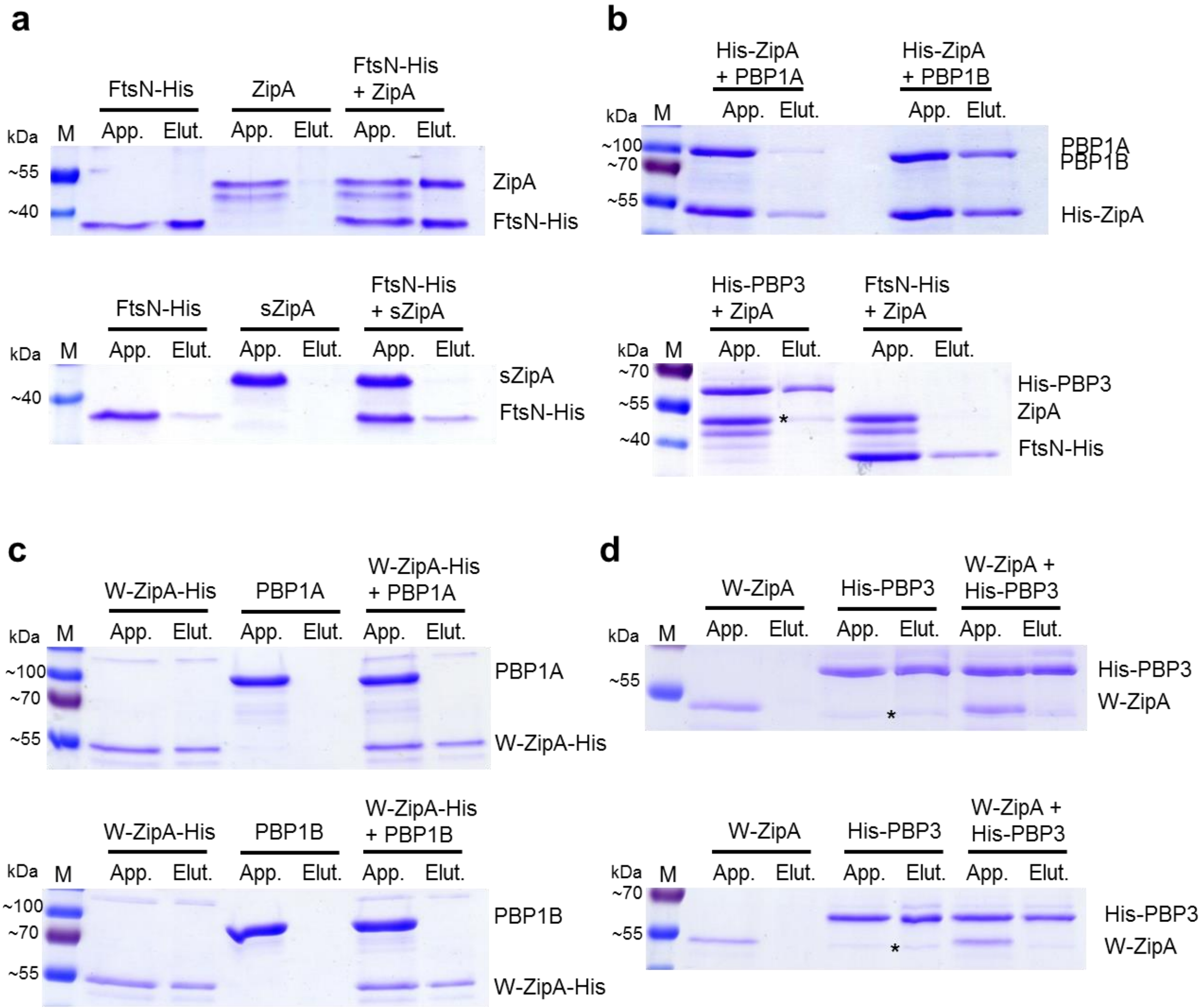

Supplementary Fig. 1. ZipA interacts with PBP1A, PBP1B and PBP3 but not with FtsN (related to Fig. 1). The interactions are assayed using purified proteins in a cross-linking/pulldown experiment, in which FtsN-His is incubated with either ZipA or sZipA in presence of 0.05% Triton X-100 (**a**); His-ZipA is incubated with either PBP1A or PBP1B in presence of 1% Triton X-100 (**b**, upper gel); and His-PBP3 or FtsN-His are incubated with ZipA in the presence of 1% Triton X-100 (**b**, lower gel). Although in 0.05% Triton X-100 FtsN-His retains ZipA (but not sZipA), this interaction does not take place when detergent is increased to 1%. The interactions between ZipA and PBP1A and PBP1B are detectable under these conditions. In the ZipA - HisPBP3 assay (panel b) the protein eluting with a similar size as ZipA is probably a degradation product of His-PBP3, which is also present in panel d (bands marked with an asterisk). **(c)** Cross-linking/pulldown experiment with PBP1A or PBP1B and W-ZipA-His (chimera protein in which the transmembrane region of ZipA is replaced by the artificial transmembrane peptide WALP23) in the presence of 1% Triton X-100. **(d)** Cross-linking/pulldown experiment with His-PBP3 and W-ZipA in the presence of 0.05% (upper gel) or 1% (bottom gel) Triton X-100. The degradation product of His-PBP3 eluted from the Ni-NTA beads, is marked with an asterisk. M, molecular weight markers; kDa, kilodalton; App., applied sample; Elut., sample eluted from Ni-NTA beads.

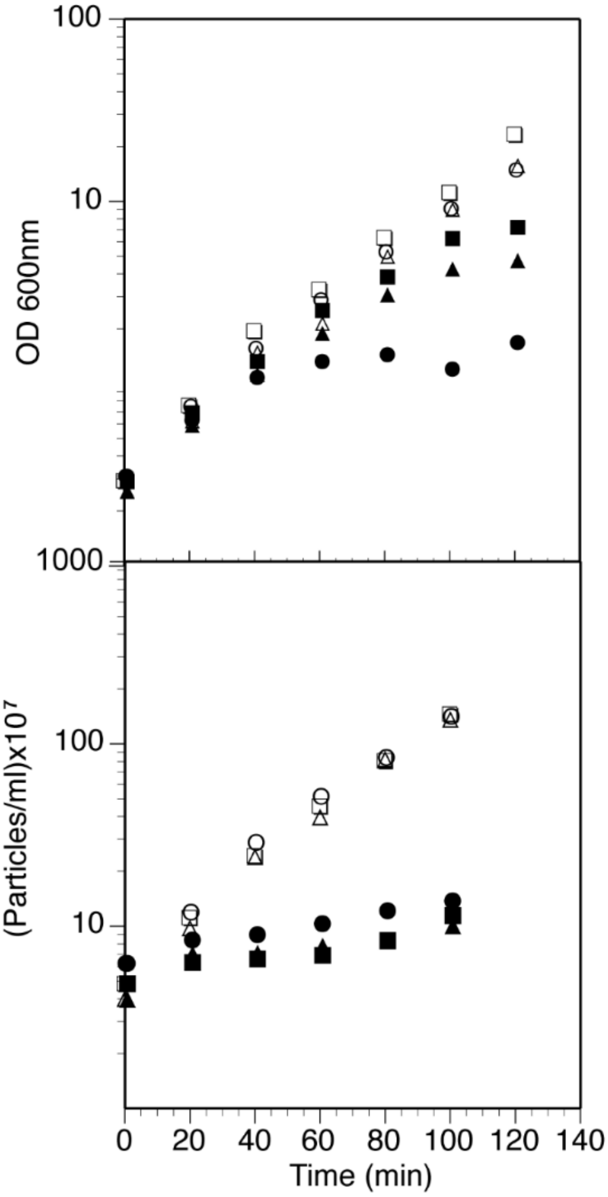

Supplementary Fig. 2. Effect of aztreonam addition on cell growth and division (related to Fig. 2). Optical density (600 nm) and particle increase were measured at the indicated time-points (plotted values corrected for dilutions) from exponential balanced growth cultures of BW25113 (square), BW25113Δ*mrcA* (triangle) and BW25113Δ*mrcB* (circle) strains in the presence (filled symbol) or absence (empty symbol) of 0.3 μg mL^{-1} of aztreonam.

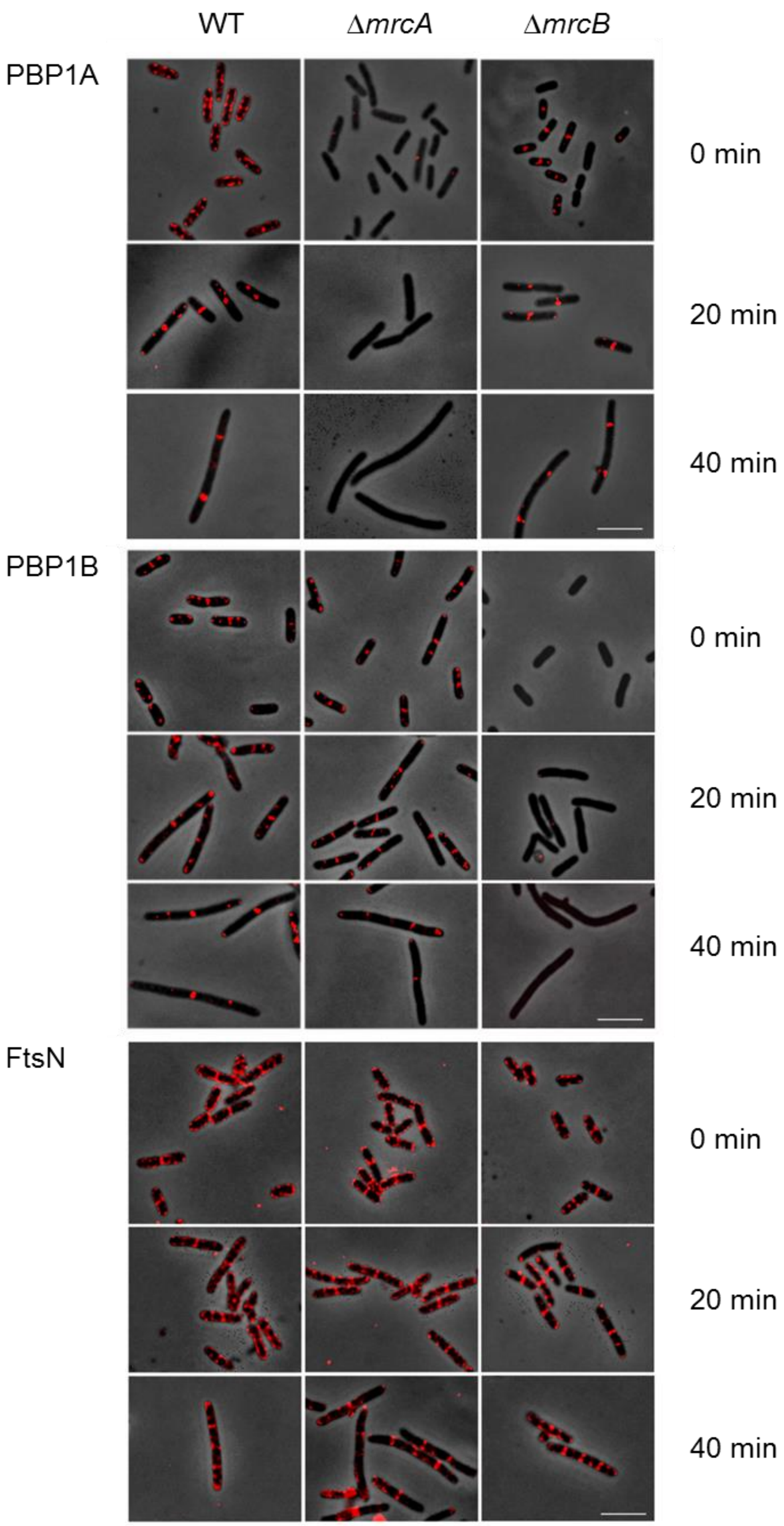
WT
ΔmrcA
ΔmrcB
PBP1A
PBP1B
FtsN
0 min
20 min
40 min
0 min
20 min
40 min
0 min
20 min
40 min

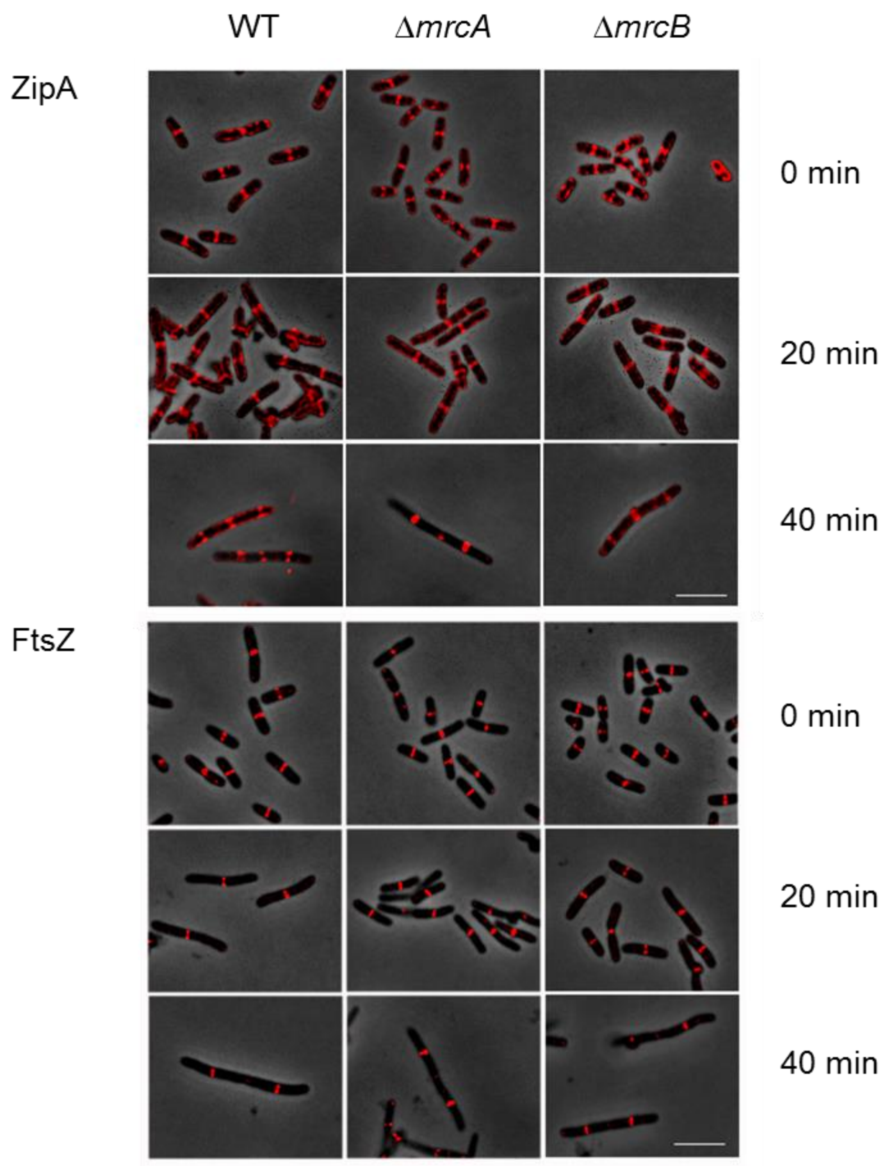

Supplementary Fig. 3. Localization of PBP1A, PBP1B, FtsN, ZipA and FtsZ during cell division inhibition by aztreonam (related to Fig. 2). Merged micrographs of phase contrast and immunolocalization images of PBP1A, PBP1B, FtsN, ZipA or FtsZ in BW25113 (WT), BW25113Δ*mrcA* (Δ*mrcA*) and BW25113Δ*mrcB* (Δ*mrcB*) cells grown in the presence of 0.3 µg mL^{-1} or absence of aztreonam (20 and 40 min). Scale bars represent 5 µm.

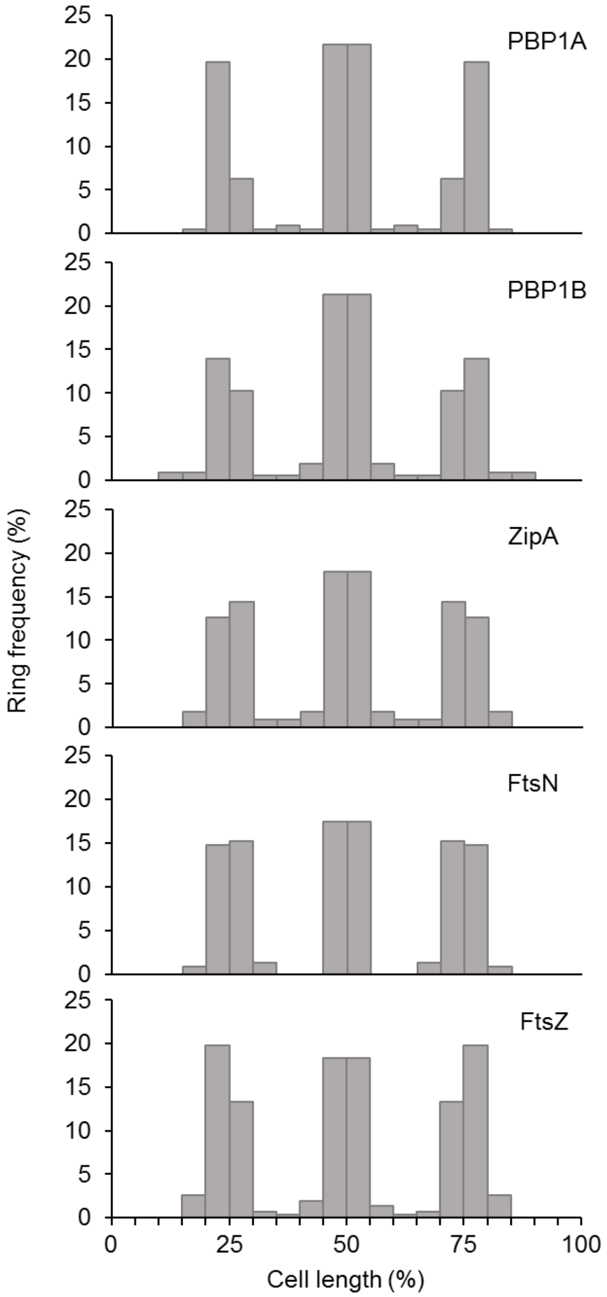

Supplementary Fig. 4. Localization of rings of PBP1A, PBP1B, FtsN, ZipA and FtsZ in WT cells grown in the presence of aztreonam (related to Fig. 2). Proteins were detected with purified antibodies in BW25113 cells grown in the presence of aztreonam for 40 min (the cells are shown in Figures 2 and S3). The distances of fluorescently labelled ring to each of the two cell poles were measured using ImageJ [1]. Both distances of each ring were normalized to the total length of the filament (set as 100%). Histograms show the distribution of rings at 5 percent intervals along the normalized length of the filaments. A total of 363 cells containing 663 rings were analysed.

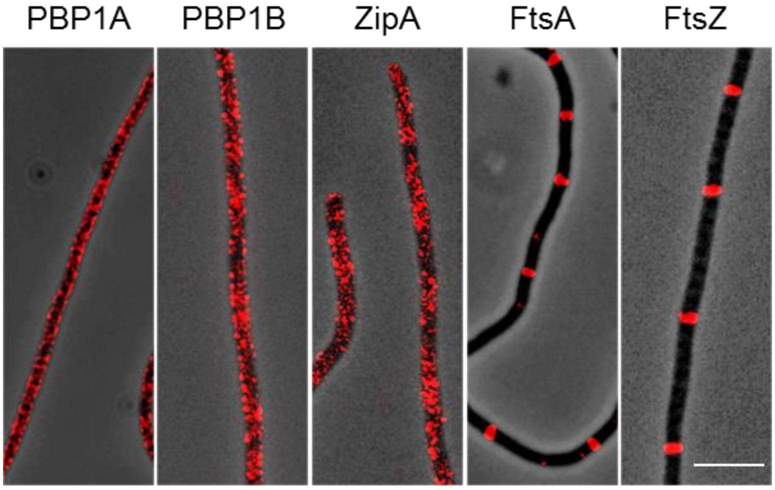

Supplementary Fig. 5. Localization of PBP1A, PBP1B, ZipA, FtsA and FtsZ in ZipA-depleted cells. Merged micrographs of phase contrast and immunolocalization images of PBP1A, PBP1B, ZipA, FtsA or FtsZ in WM1304 cells (Δ*zipA* / *rep*^{ts} *zipA*) grown at 42°C. An overnight culture grown at 30°C was diluted 1:500 in LB media, grown for 1 h at 30°C and further incubated at 42°C for 160 min. Scale bars represent 5 μm.

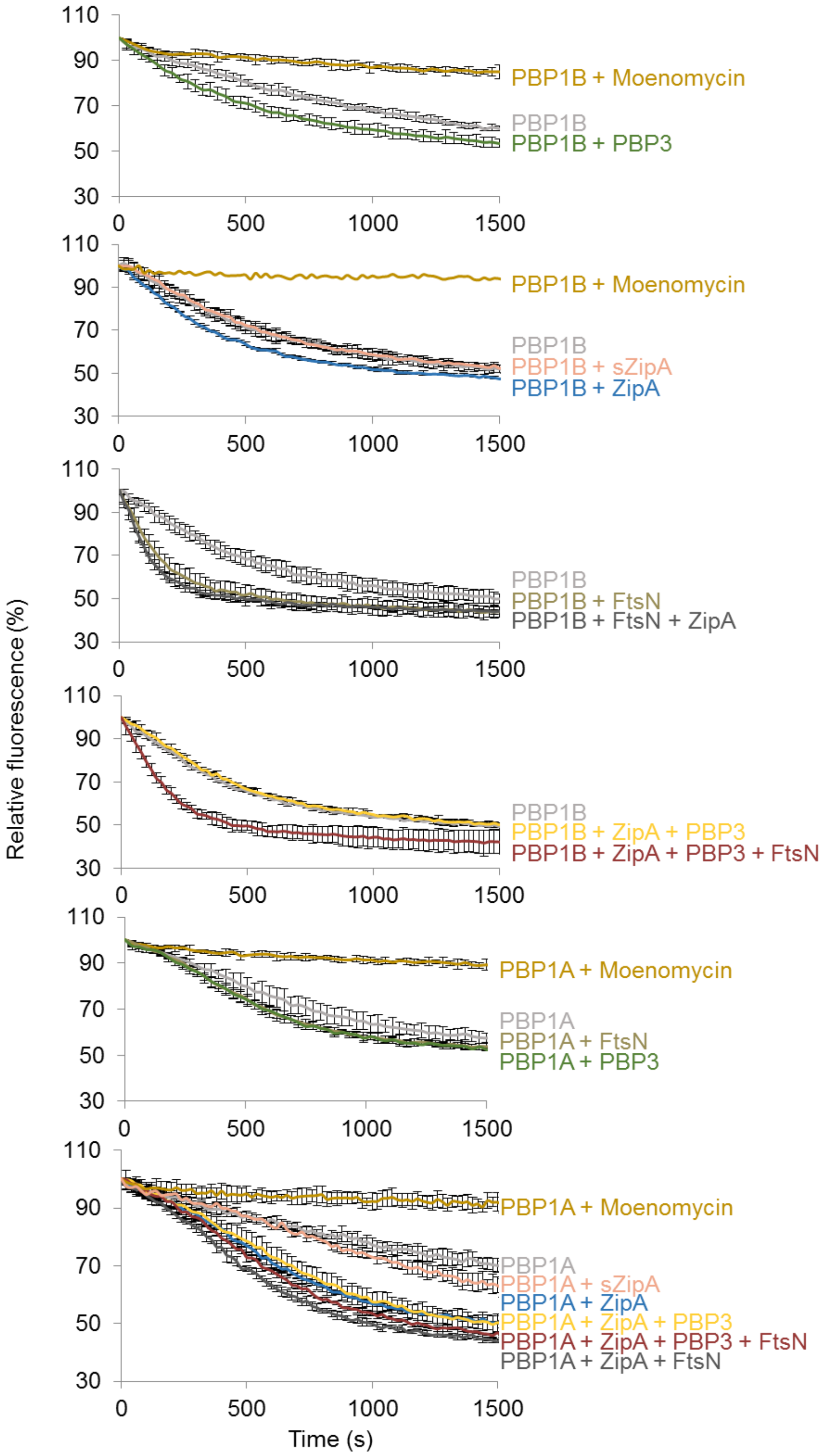

Supplementary Fig. 6. GTase activity assays for data shown in Fig. 3. Continuous fluorescence assay graphs for GTase activity of PBP1B and PBP1A. Each value is the mean ± s.d. of 3 independent experiments.

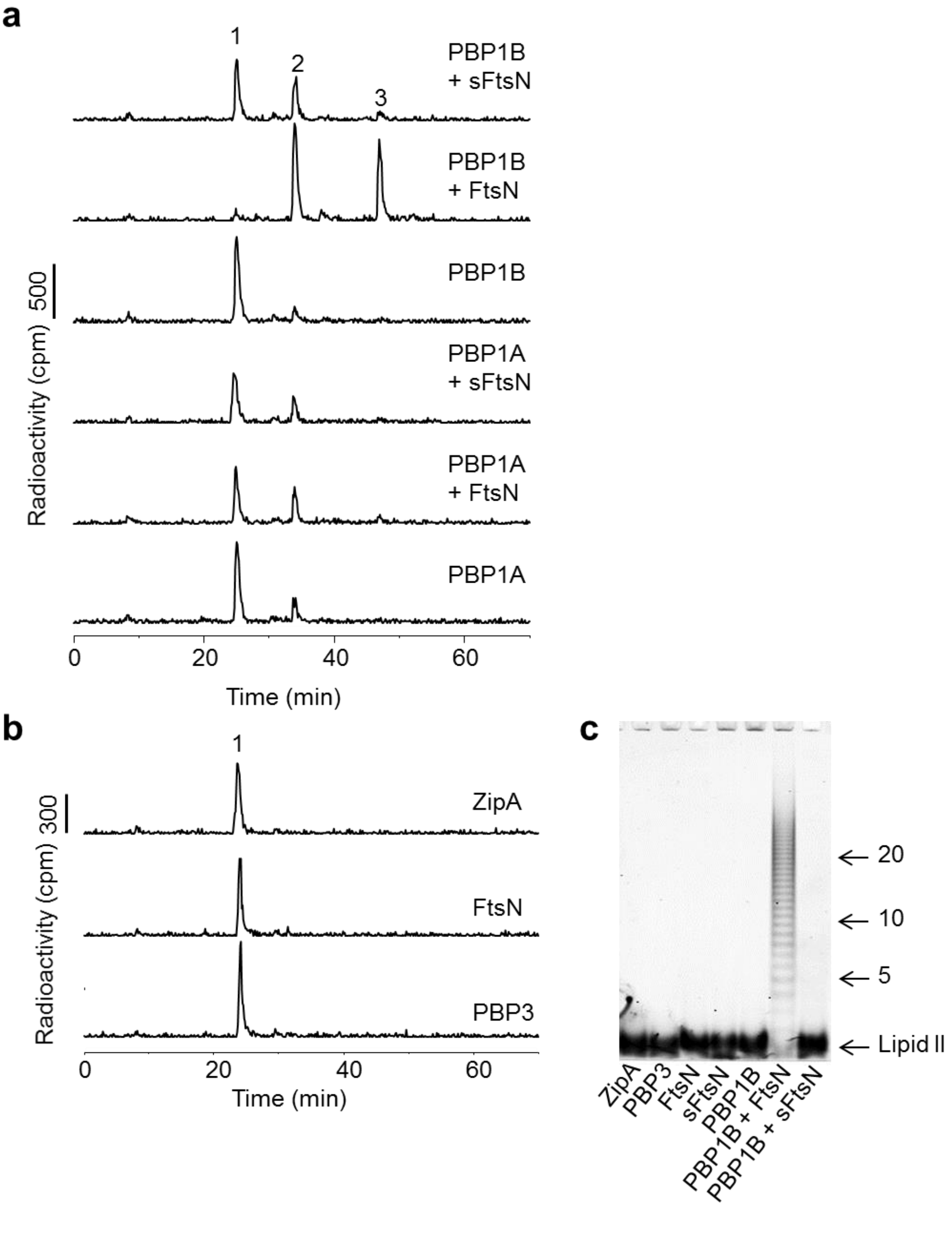

Supplementary Fig. 7. Stimulation of PBP1A and PBP1B at low concentration and control samples (related to Fig. 4). (a) Representative HPLC chromatograms of *in vitro* PG synthesis reactions in the presence of the indicated proteins using radioactive lipid II as substrate. The synthesised PG was digested with cellosyl, reduced with sodium borohydride and analysed by HPLC. Peak 1 is generated from glycan chain ends and unreacted lipid II, peak 2 is a GTase product and peak 3 is a GTase/TPase product (see structures in Figure 4c). **(b and c)** Control reactions showing that the purified preparations of ZipA, FtsN, PBP3 and sFtsN are inactive in *in vitro* PG synthesis reactions (related to Figure 4).

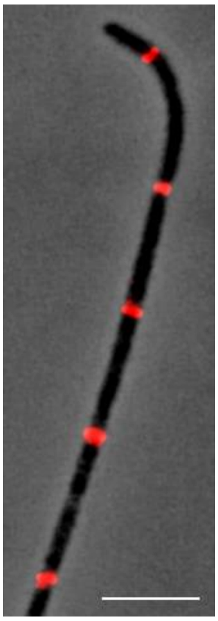

Supplementary Fig. 8. Localization of FtsZ in cells depleted of ZipA and FtsN (related to Fig. 5). Merged micrograph of phase contrast and immunolocalization image of FtsZ in MPW29 cells (*ftsA*^{E124A} Δ*zipA* Δ*ftsN/ rep*^{ts} *zipA ftsN*) grown at 42°C. An overnight culture grown at 30°C was diluted 1:500 in LB media, grown for 1 h at 30°C and further incubated at 42°C for 160 min. Scale bar represent 5 µm.

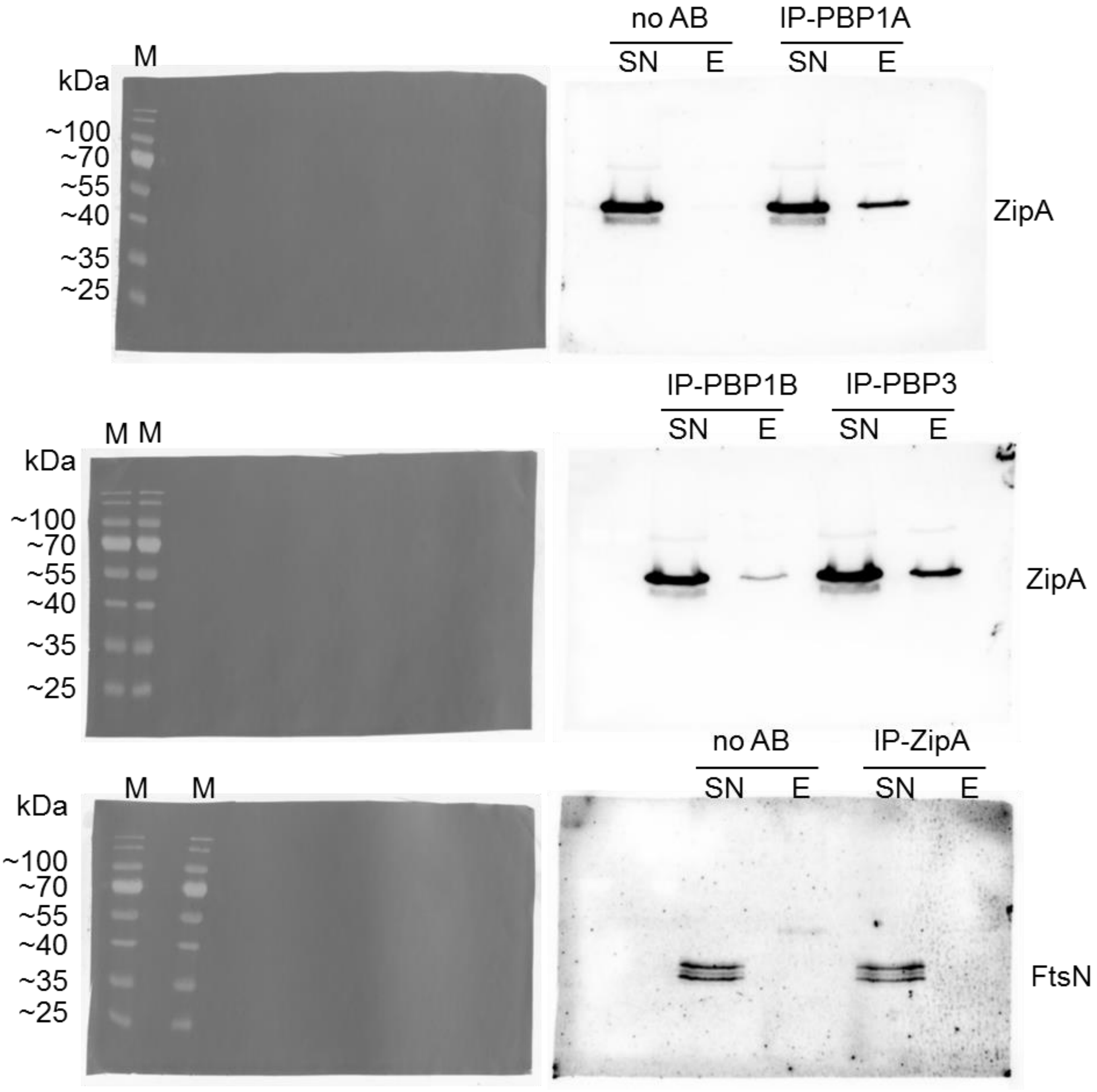

Supplementary Fig. 9. Uncropped pictures of the Western Blots shown in Fig. 1b. Membrane (left) and fluorescence (right) pictures of the western blots performed for *in vivo* co-immunoprecipitation assays. M, molecular weight marker; kDa, kilodalton.

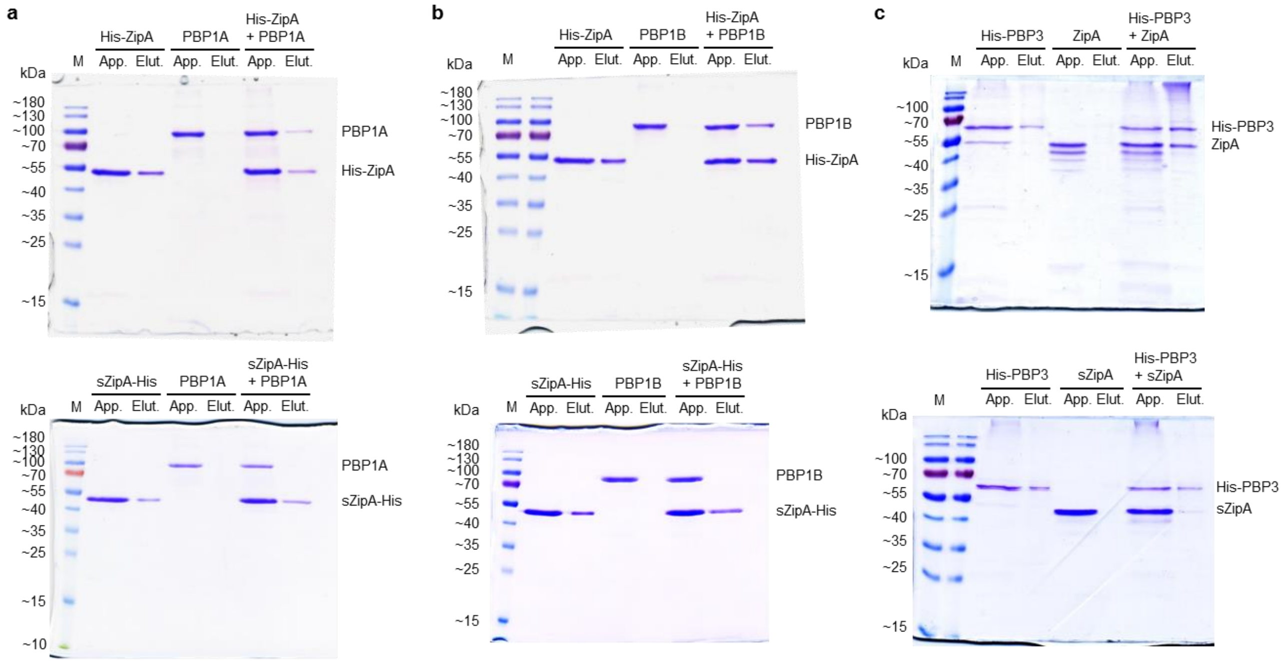

Supplementary Fig. 10. Uncropped pictures of the gels shown in Fig. 1c, 1d and 1e. M, molecular weight markers; kDa, kilodalton; App., applied sample; Elut., sample eluted from Ni-NTA beads.

Supplementary Table 1. Quantification of preseptal PG synthesis bands.

Strain	Total cell length measured (µm)	Total No. of preseptal PG synthesis bands	Preseptal PG bands per µm
**WM2935**	4867.11	453	0.093
**MPW22**	5214.47	389	0.075
**MPW23**	6072.33	566	0.093
**MPW29**	24622.52	438	0.018
**MPW30**	12835.57	200	0.016

Supplementary Table 2. Strains and plasmids.

Strain or plasmid	Genotype / properties	Source or reference
**Strain**		
BL21(DE3)	Expression strain	Novagen
BL21(DE3)pLysS /pET-15Zip	Overexpression of His-ZipA	[2]
BW25113	Wild type strain	[3]
BW25113 Δ*mrcA*	BW25113 *mrcA::aph*	[4]
BW25113 Δ*mrcB*	BW25113 *mrcB::aph*	[4]
LOBSTR	Expression strain	[5] (Kerafast)
MPW22	WM2935 *zipA*::*aph* / pCH32	This work
MPW23	WM2935 *ftsN*::*cat* / pWM2245	This work
MPW29	WM2935 *zipA*::*aph ftsN*::*cat* / pCH32 pWM2245	This work
MPW30	WM2935 *zipA*::*aph ftsN*::*cat /* pCH32	This work
WM1304	MG1655 *lacU169 zipA*::*aph /* pCH32	(W. Margolin collection)
WM2245	W3110 / pWM2245	[6]
WM2355	W3110 *argE*::Tn*10 ftsN*::*cat* / pWM2245	[6]
WM2935	W3110 *leu*::Tn*10 ftsA (E124A)*	[6]
**Plasmid**		
pCH32	*rep*^{ts}, *zipA*, *ftsZ,* Spc^{R}	[7]
pDML924	pET28a(+) encoding His-PBP1Bγ	[8]
pET-15Zip	pET15b encoding His-ZipA	[2]
pET28-His-LpoA(sol)	pET28a(+) encoding His-LpoAΔ1-27	[9]
pET28-His-LpoB(sol)	pET28a(+) encoding His-LpoBΔ1-20	[9]
pFE42	pHis17 encoding FtsN-His	[10]
pHis17-ECN2	pHis17 encoding FtsNΔ1-57-His	[10]
pMVR1	pQE30 encoding His-PBP3	[11]
pPZW05	pET28a(+) encoding ZipA-His	This work
pPZW06	pET28a(+) encoding ZipAΔ1-25-His	This work
pPZW22	pET28a(+) encoding WALP23-ZipAΔ1-25-His	This work
pTK1Ahis	pET28a(+) encoding His-PBP1A	[12]
pWM2245	*rep*^{ts}, *ftsN,* Ap^{R}	[6]

Supplementary Table 3. Quantification of rings.

Strain	Time after aztreonam (min)	% of cells showing rings containing				
		PBP1A	PBP1B	FtsN	FtsZ	ZipA
**WT**	0	77	97	89	98	98
	20	73	80	87	96	97
	40	71	75	84	94	95
**Δ*mrcA***	0	5	92	81	98	98
	20	6	91	80	93	97
	40	4	71	77	92	96
**Δ*mrcB***	0	85	0	85	98	98
	20	82	1	84	97	97
	40	73	0	82	96	96

Supplementary References

1 Schneider, C. A., Rasband, W. S. & Eliceiri, K. W. NIH Image to ImageJ: 25 years of image analysis. *Nat Meth* **9**, 671-675 (2012).

2 RayChaudhuri, D. ZipA is a MAP-Tau homolog and is essential for structural integrity of the cytokinetic FtsZ ring during bacterial cell division. *EMBO J.* **18**, 2372-2383 (1999).

3 Datsenko, K. A. & Wanner, B. L. One-step inactivation of chromosomal genes in *Escherichia coli* K-12 using PCR products. *Proc. Nat. Acad. Sci. USA* **97**, 6640-6645 (2000).

4 Baba, T. *et al.* Construction of *Escherichia coli* K-12 in-frame, single-gene knockout mutants: the Keio collection. *Mol. Sys. Biol.* **2**, 2006 0008, doi:10.1038/msb4100050 (2006).

5 Andersen, K. R., Leksa, N. C. & Schwartz, T. U. Optimized *E. coli* expression strain LOBSTR eliminates common contaminants from His-tag purification. *Proteins* **81**, 1857-1861 (2013).

6 Bernard, C. S., Sadasivam, M., Shiomi, D. & Margolin, W. An altered FtsA can compensate for the loss of essential cell division protein FtsN in *Escherichia coli*. *Mol. Microbiol.* **64**, 1289-1305 (2007).

7 Hale, C. A. & de Boer, P. A. Direct binding of FtsZ to ZipA, an essential component of the septal ring structure that mediates cell division in *E. coli*. *Cell* **88**, 175-185 (1997).

8 Terrak, M. *et al.* The catalytic, glycosyl transferase and acyl transferase modules of the cell wall peptidoglycan-polymerizing penicillin-binding protein 1b of *Escherichia coli*. *Mol Microbiol* **34**, 350-364 (1999).

9 Typas, A. *et al.* Regulation of peptidoglycan synthesis by outer-membrane proteins. *Cell* **143**, 1097-1109 (2010).

10 Yang, J. C., Van Den Ent, F., Neuhaus, D., Brevier, J. & Löwe, J. Solution structure and domain architecture of the divisome protein FtsN. *Mol. Microbiol* **52**, 651-660 (2004).

11 Bertsche, U. *et al.* Interaction between two murein (peptidoglycan) synthases, PBP3 and PBP1B, in *Escherichia coli*. *Mol. Microbiol.* **61**, 675-690 (2006).

12 Born, P., Breukink, E. & Vollmer, W. *In vitro* synthesis of cross-linked murein and its attachment to sacculi by PBP1A from *Escherichia coli*. *J. Biol. Chem.* **281**, 26985-26993 (2006).
